# Duraplasty Versus Bony Decompression in Adult Chiari I: Comparative Clinical and Morphometric Analysis

**DOI:** 10.3390/medicina62061076

**Published:** 2026-06-01

**Authors:** Recai Engin, Fatih Tomakin, Hasan Şener, Gürkan Gökalp, Vaner Köksal, Şevki Serhat Baydın, Mustafa Aras, Cengiz Çokluk

**Affiliations:** 1 Department of Neurosurgery, Faculty of Medicine, Samsun University, 55270 Samsun, Türkiye; fatih.tomakin@samsun.edu.tr (F.T.); vaner.koksal@samsun.edu.tr (V.K.); 2 Department of Neurosurgery, Ankara Gazi Mustafa Kemal Occupational and Environmental Diseases Hospital, 06560 Ankara, Türkiye; hasansener1989@gmail.com; 3 Department of Neurosurgery, Faculty of Medicine, Ondokuz Mayıs University, 55270 Samsun, Türkiye; gurkangokalp55@gmail.com (G.G.); drsserhatb@gmail.com (Ş.S.B.); mustafa.aras@omu.edu.tr (M.A.); cengizcokluk@yahoo.com (C.Ç.)

**Keywords:** Chiari malformation type I, suboccipital decompression, duraplasty, syringomyelia, Chicago Chiari outcome scale, morphometric analysis, foramen magnum

## Abstract

*Background and Objectives*: The optimal surgical technique for Chiari type I malformation (CM-I) remains debated, particularly in patients without syringomyelia. While duraplasty (DP+) may enhance radiological outcomes, it can carry higher complication risks. We compared clinical, radiological, and morphometric outcomes after suboccipital decompression with DP+ and without duraplasty (DP−), with prespecified subgroup analyses by syringomyelia status. *Materials and Methods*: Ninety-three consecutive adult CM-I underwent DP− (n = 54) or DP+ (n = 39) between 2014 and 2022. Pre- and post-operative MRI and neurological evaluations were obtained at 1 year. Functional recovery was assessed using the Chicago Chiari Outcome Scale (CCOS). Clinical and radiological outcomes, complication rates, and subgroup results (with vs. without syringomyelia) were compared. *Results*: Overall clinical improvement was observed in 92.5% of patients (DP−: 94.4%; DP+: 89.7%; *p* > 0.05). Among patients without syringomyelia, clinical improvement remained high with DP−. Radiological benefit—including syrinx regression and mega cisterna magna formation—was greater with DP+ (64.1% vs. 24.1%; *p* < 0.001), but this did not translate into improved functional outcomes (*p* > 0.05). Cerebellar slump occurred more often after DP+ (30.8% vs. 9.3%; *p* < 0.05). Complication rates, particularly CSF-related events, were significantly higher with DP+ (17.9% vs. 3.7%, *p* = 0.032). Morphometric expansion of the foramen magnum did not correlate with functional outcomes. *Conclusions*: At 1-year follow-up, suboccipital decompression without duraplasty appears to provide comparable clinical improvement to DP+, with fewer complications, in selected CM-I patients without syringomyelia. Duraplasty offers radiological advantages, especially for syringomyelia, but at the cost of increased risk. Longer follow-up is necessary to determine the durability of these findings.

## 1. Introduction

Chiari type I malformation (CM-I) is defined as caudal displacement of the cerebellar tonsils through the foramen magnum, frequently associated with altered cerebrospinal fluid (CSF) dynamics and, in many cases, syringomyelia [1,2,3]. Clinical presentation is heterogeneous, ranging from headache to cranial nerve dysfunction, and may include syrinx-related symptoms such as motor weakness and sensory changes [4,5]. Recent systematic reviews have also highlighted advances in the epidemiology and pathogenesis of CM-I, underlining its complex and multifactorial nature [2].

The optimal surgical strategy for CM-I remains controversial. Posterior fossa decompression without duraplasty (DP−) is considered less invasive, with shorter operative time and lower risk of CSF-related complications [6,7,8,9], whereas decompression with duraplasty (DP+) is thought to achieve more effective restoration of CSF pathways and higher rates of syrinx regression [10,11,12]. A large multicenter analysis from the Park-Reeves Syringomyelia Research Consortium demonstrated that surgical technique, particularly the use of duraplasty, significantly influences both complication rates and long-term outcomes in pediatric patients [13].

Several systematic reviews and meta-analyses have attempted to clarify the relative benefits of these techniques, but the results remain inconsistent [10,11,14,15]. Consensus recommendations and expert opinion documents have emphasized tailoring the surgical approach to individual patient characteristics, including the presence of syringomyelia and the degree of tonsillar descent [16,17]. Yılmaz et al. proposed a tonsillar-descending grading scale to guide the choice of duraplasty, underscoring the role of morphometric assessment in surgical decision-making [18].

Clinical outcomes also vary widely across reports. Mueller and Oro found that presenting symptoms can differ substantially between patients with and without syringomyelia [4]. Pepper et al. reported heterogeneous improvement rates (27–88%) in non-syringomyelia patients following decompression [19], while Giammattei et al. observed long-term improvement in 87% [20], and Beretta et al. noted 93% improvement in Chiari-related headache [21]. Yu et al. recently reported that posterior fossa decompression with duraplasty yielded significantly higher CCOS scores compared with bone-only decompression in a cohort of non-syringomyelia patients [22].

Durability of results remains an important issue. Lin et al. documented reoperation rates of 31.9% after DP− at long-term follow-up [9], whereas Tubbs et al. and Alfieri & Pinna reported markedly lower rates (0–3.2%) after DP+ [23,24]. Mozaffari et al. confirmed that long-term failures are more frequent after DP−, particularly in patients with persistent or progressive syringomyelia [25]. Recent pediatric series by Hale et al. [26] and El-Hajj et al. [27] emphasized the prognostic role of syrinx size and confirmed that complication rates remain broadly comparable between DP− and DP+ in high-volume cohorts.

Morphometric studies have further suggested that posterior cranial fossa dimensions and syrinx characteristics influence both radiological and clinical outcomes, highlighting the importance of individualized patient selection [18,28].

In this study, we aimed to compare clinical, radiological, and morphometric outcomes of DP+ versus DP− in adult CM-I patients, with subgroup analyses by syringomyelia status and age. Functional outcomes were assessed using the Chicago Chiari Outcome Scale (CCOS), which provides disease-specific evaluation superior to generic disability scales. Our hypothesis was that suboccipital decompression without duraplasty may achieve comparable short-term clinical improvement in selected patients without syringomyelia.

## 2. Materials and Methods

### 2.1. Ethical Approval

Ethical approval for this study was obtained from the Ondokuz Mayıs University Clinical Research Ethics Committee (Approval No: 2024/571). The study was conducted in accordance with the principles of the Declaration of Helsinki. Written informed consent was obtained from all participants.

### 2.2. Patient Selection and Inclusion Criteria

This single-center, retrospective, consecutive cohort included adult patients with Chiari type I malformation treated between 2014 and 2022. A total of 100 consecutive patients were screened; 7 were excluded due to prior Chiari surgery (either at our institution or elsewhere), non-Chiari I subtypes, or incomplete 1-year radiological follow-up, leaving 93 patients for analysis. Inclusion criteria were age ≥18 years, radiologically confirmed CM-I, and treatment with suboccipital decompression with (DP+) or without duraplasty (DP−), with available baseline and 1-year post-operative clinical and MRI data. Exclusion criteria were other Chiari subtypes, incomplete clinical or radiological data, prior CM-I surgery at any institution, or absence of 1-year post-operative imaging. Presence or absence of syringomyelia was recorded, but was neither an inclusion/exclusion criterion nor the sole determinant of surgical technique; the rationale for DP+ vs. DP− is detailed in the Surgical Technique subsection. Because only 7% of screened patients were excluded, a formal baseline comparison between excluded and included patients was not performed.

### 2.3. Radiological Evaluation and Data Collection

Demographic data, including age and sex, were recorded. Pre-operative spinal MRI was reviewed for the presence of syringomyelia. The degree of tonsillar herniation was measured from the McRae line and classified as follows: Grade 1 (5–9 mm), Grade 2 (10–14 mm), Grade 3 (15–19 mm), and Grade 4 (>19 mm). Tonsillar herniation was measured preoperatively using the McRae line on sagittal MRI. For post-operative assessment of cerebellar descent (cerebellar slump), the McGregor line was used, as suboccipital decompression alters the posterior margin of the foramen magnum, potentially limiting the reliability of the McRae line in post-operative measurements. Pre- and post-operative MRI at 1 year was used to assess syringomyelia regression, mega cisterna magna formation, tonsillar descent, and cerebellar slump. Cerebellar slump was defined as cerebellar descent below the McGregor line greater than the pre-operative tonsillar herniation.

**Figure 1:**
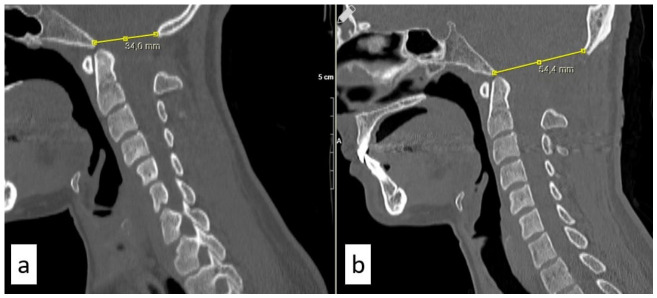
Representative sagittal CT images demonstrating the measurement of the anteroposterior diameter of the foramen magnum at the McRae line. (**a**) Pre-operative image showing the foramen magnum diameter, and (**b**) post-operative image demonstrating the new foramen magnum diameter following suboccipital decompression, measured in millimeters. Both images are from the same patient. The McRae line is defined between the basion and opisthion, and the anteroposterior diameter was measured along this line.

Morphometric analysis included measurement of the anteroposterior diameter of the foramen magnum at the McRae line, both preoperatively and postoperatively. The measurement technique is illustrated in Figure 1 using a representative sagittal CT image.

### 2.4. Clinical Outcome Assessment

At the 1-year post-operative follow-up, clinical symptoms and MRI findings were reevaluated. Patients who demonstrated a marked reduction in symptoms were considered to have achieved clinical benefit. Radiological benefit was defined as the presence of one or more of the following findings on 1-year post-operative MRI: reduction in tonsillar herniation, formation of a mega cisterna magna (MCM), or resolution/reduction of syringomyelia. Surgical complications, including cerebrospinal fluid (CSF) leakage, wound infection, and meningitis, were identified from operative notes and follow-up records.

Functional outcomes were assessed using the Chicago Chiari Outcome Scale (CCOS). CCOS scores were determined at the 1-year follow-up based on systematically documented clinical records and standardized follow-up evaluations. The CCOS is a validated scoring system ranging from 4 to 16 points, incorporating pain symptoms, non-pain symptoms, functional status, and complications. Importantly, CCOS scoring was performed using predefined criteria based on recorded clinical data rather than subjective estimation. Clinical benefit, as defined above, was evaluated independently of CCOS scores and was not derived from a specific CCOS threshold.

### 2.5. Surgical Technique and Group Allocation

All patients underwent suboccipital decompression. Among patients with syringomyelia (n = 50), 25 received DP+ and 25 received DP−; among those without syringomyelia (n = 43), 14 received DP+, and 29 received DP−. The decision to perform duraplasty in non-syringomyelia cases was individualized based on pre-operative morphometry (e.g., degree of tonsillar descent, posterior fossa dimensions) and intraoperative findings (dural pulsatility).

### 2.6. Bony Decompression

In both groups, a midline suboccipital craniectomy of approximately 2.5 × 2.5 cm was performed, followed by a C1 laminectomy. The laminectomy extended ~2 cm laterally from the midline on each side, in line with anatomical and surgical series emphasizing safe decompressive margins without endangering the vertebral arteries or atlanto-axial stability [13,20].

### 2.7. Dural Management

In DP−, the inner dural layer was left intact; the outer (parietal) leaf underwent limited “keyhole” dura-splitting incisions to reduce scarring and optimize decompression. In DP+, a Y-shaped durotomy was performed, and the dura was expanded with a watertight graft using either autologous pericranium or a synthetic patch.

In all cases, intraoperative ultrasound and/or direct visualization were used to confirm restoration of CSF pulsation at the foramen magnum. Meticulous hemostasis was achieved in every procedure. Closure was performed in anatomical layers, with particular attention to muscle reapproximation and subgaleal drainage when indicated. Post-operative management was standardized across both groups.

### 2.8. Statistical Analysis

The primary outcome measure of this study was the 12-month CCOS. Continuous variables were compared with independent *t*-tests (or Mann–Whitney U if non-normal; normality by Shapiro–Wilk); categorical outcomes with Pearson χ^2^ (or Fisher’s exact when expected counts <5). CCOS scores were additionally analyzed using the Mann–Whitney U test, given their ordinal nature. Within-group morphometry (pre/post) used paired *t*-tests (or Wilcoxon), and correlations involving CCOS were assessed using Spearman’s rank correlation coefficient due to the ordinal nature of the data. All tests were two-sided (α = 0.05); no multiplicity adjustment. Table 1 is descriptive only. Analyses were complete-case and conducted in SPSS v26 (IBM, Armonk, NY, USA).

## 3. Results

### 3.1. Study Cohort and Allocation

Of 100 consecutive adults screened (2014–2022), 7 were excluded (prior Chiari surgery, non-CM-I subtype, or incomplete 1-year imaging), leaving 93 patients for analysis. Surgical allocation was DP+ (n = 39) and DP− (n = 54). Baseline characteristics are summarized in Table 1. The prevalence of syringomyelia at baseline did not differ by technique (DP+ 25/39 [64.1%] vs. DP− 25/54 [46.3%], χ^2^ = 2.888, *p* = 0.089; Table 2).

### 3.2. Illustrative Adult Cases

**Figure 2:**
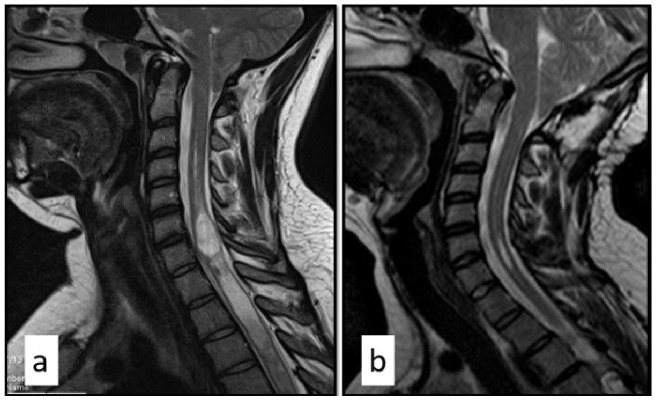
(Illustrative case 1) A 19-year-old male patient presenting with headaches and numbness in the arms, particularly during coughing and sneezing. (**a**) Pre-operative sagittal T2-weighted MRI demonstrates cerebellar tonsillar herniation measuring 14 mm (Grade 2) and syringomyelia extending from the C3 level to the thoracic spine. (**b**) One-year post-operative sagittal T2-weighted MRI following DP− shows a significant reduction in syringomyelia.

**Figure 3:**
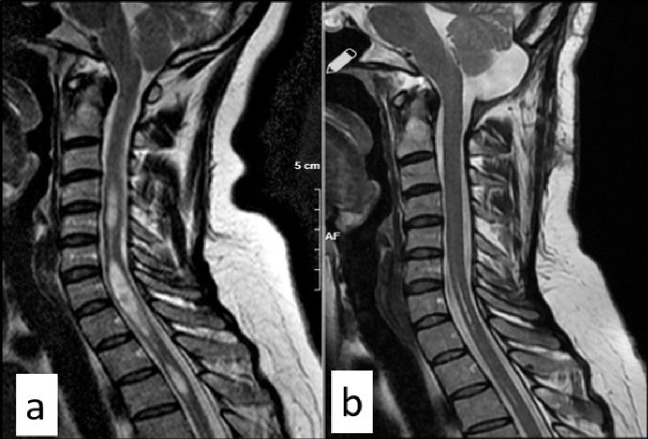
(Illustrative case 2) A 45-year-old female patient presenting with headache and dysphagia. (**a**) Pre-operative sagittal T2-weighted MRI demonstrates cerebellar tonsillar herniation measuring 6 mm (Grade 1) and syringomyelia extending from the C4 level to the T3 level. (**b**) One-year follow-up sagittal T2-weighted MRI after DP+ shows significant reduction of syringomyelia and formation of mega cisterna magna (MCM).

Both surgical methods applied for Chiari type 1 disorder are shown in illustrative cases (Figure 2 and Figure 3).

### 3.3. Radiological Outcomes

Composite radiological benefit was more frequent after duraplasty (DP+ 25/39 [64.1%] vs. DP− 13/54 [24.1%], χ^2^ = 15.015, *p* < 0.001 ). Formation of a mega cisterna magna by 1 year paralleled this pattern (Table 2) and was not associated with clinical benefit (Table 3). Regression of tonsillar herniation did not differ between groups (Table 2).

### 3.4. Clinical Outcomes

At 12 months, clinical improvement exceeded 90% in both groups (DP+ 35/39 [89.7%] vs. DP− 51/54 [94.4%], *p* = 0.448, Fisher’s exact test; Table 4), with no significant difference between groups. However, CSF-related complications were significantly more frequent in the DP+ group (17.9% vs. 3.7%, *p* = 0.032; Table 4). Mean CCOS scores were similar (DP− 13.77 ± 2.31 vs. DP+ 13.00 ± 2.55, *p* = 0.129; Table 5A). When stratified by syringomyelia status, CCOS scores also did not differ significantly (no syrinx 13.79 ± 2.09 vs. syrinx 13.16 ± 2.67, *p* = 0.215; Table 5B), indicating consistent functional outcomes regardless of syrinx presence.

### 3.5. Relationship Between Postoperative MCM Formation and Clinical Improvement

At 1 year, MCM was observed in 38/93 patients (40.9%). When stratified by MCM status, clinical improvement was similar (94.7% with MCM vs. 90.9% without MCM), and the difference was not statistically significant (Fisher’s exact test, *p* = 0.71) (Table 3). These findings indicate that post-operative MCM formation does not reliably predict functional recovery.

### 3.6. Complications

CSF-related events (including closed pseudomeningocele) occurred more often with duraplasty (DP+ 7/39 [17.9%] vs. DP− 2/54 [3.7%], Fisher’s exact test, *p* = 0.032; Table 4). Overall complication rates were similar between groups (DP+ 9/39 [23.1%] vs. DP− 9/54 [16.7%], *p* = 0.440; Table 4). Cerebellar slump—defined morphometrically relative to the McGregor line—was more common after DP+ than DP− (30.8% vs. 9.3%, *p* = 0.008; Table 2).

### 3.7. Subgroup and Sensitivity Analyses

Morphometric analysis demonstrated a significant increase in foramen magnum diameter in both groups, with no between-group difference in the magnitude of expansion (Table 6). Findings were consistent in analyses stratified by syringomyelia status (Table 5B).

### 3.8. Morphometric Findings

Post-operative foramen magnum diameter increased significantly in both groups. However, Spearman correlation analysis demonstrated no significant association between foramen magnum diameter and functional outcome at 1 year. Specifically, neither pre-operative (r = −0.041, *p* = 0.696) nor post-operative (r = 0.017, *p* = 0.872) foramen magnum diameters correlated with CCOS scores (Table 7). These findings indicate that morphometric expansion alone does not translate into functional recovery. The near-zero correlation coefficients further support a dissociation between anatomical decompression and clinical outcome.

## 4. Discussion

In this study, we demonstrated that both DP+ and DP− yielded high rates of clinical improvement at 1 year, with no significant difference in CCOS outcomes. In our cohort, mean CCOS was 13.77 ± 2.31 for DP− vs. 13.00 ± 2.55 for DP+ (*p* = 0.129), and ≥1-point clinical improvement occurred in >90% of patients in both groups. This is a key finding, as it suggests that bone-only decompression may provide sufficient short-term clinical benefit in carefully selected CM-I patients without syringomyelia. By applying the CCOS scale, our results provide disease-specific functional insight not captured by generic disability measures [22]. Several studies have reported that in selected cases, suboccipital decompression without duraplasty (DP−) may yield satisfactory outcomes, but few have objectively compared both techniques in matched cohorts using clinical and radiological metrics.

Radiological outcomes, however, clearly favored DP+. Overall radiological benefit was 64.1% with DP+ vs. 24.1% with DP− (*p* < 0.001), and mega cisterna magna formation was more frequent after DP+. Our data showed that syrinx regression and mega cisterna magna formation were significantly more frequent in the DP+ group. This observation aligns with prior studies demonstrating radiological superiority of duraplasty [10,11,12,29], but our work also highlights the dissociation between radiological and clinical outcomes. Similar discrepancies were noted by Pepper et al. [19], Giammattei et al. [20], and Beretta et al. [21], where radiological changes did not always correlate with symptom improvement. Thus, our findings support the notion that radiological benefit should not be equated with guaranteed clinical advantage. To better contextualize our findings within the existing literature, a comparative summary of key studies evaluating surgical outcomes in CM-I is presented (Table 8). While several studies favor duraplasty in terms of radiological improvement, consistent superiority in clinical outcomes has not been demonstrated. As summarized in Table 8, prior studies consistently demonstrate that duraplasty provides superior radiological outcomes, particularly in terms of syrinx regression, whereas its impact on clinical improvement remains variable. While several large series and meta-analyses report slightly higher clinical success rates with duraplasty, this advantage is often accompanied by increased complication rates [8,9,10]. Conversely, bone-only decompression has been associated with comparable clinical outcomes in selected patient groups, particularly those without syringomyelia, but with a more favorable safety profile [6,8,13]. These findings collectively support our observation that radiological superiority does not necessarily translate into better functional outcomes and highlight the importance of individualized surgical decision-making.

Morphometric analysis revealed significant expansion of the foramen magnum in both groups, yet this change did not correlate with CCOS improvement. This aligns with Bogdanov et al. [28], who showed that morphometric variables alone are insufficient predictors of outcome, reinforcing that symptomatology and functional status should guide surgical decision-making rather than radiological thresholds alone.

Cerebellar slump occurred more often in the DP+ group in our series, despite standardized decompression size (2.5 × 2.5 cm). We defined slump as descent below the McGregor line greater than pre-operative tonsillar herniation, a morphometric definition not uniformly reported in the literature. This finding contrasts with reports suggesting minimal slump when decompression is limited [18]. Our results, therefore, raise the possibility that individual cranial morphology, rather than decompression size alone, may predispose to slump.

Complication rates in our cohort were higher after DP+, particularly for CSF fistulas and pseudomeningocele. CSF fistula/pseudomeningocele developed in 7/39 (17.9%) with DP+ versus 2/54 (3.7%) with DP− (*p* = 0.032), indicating a significantly higher rate of CSF-related complications in the DP+ group, noting that we counted closed pseudomeningocele within CSF-related events. Our leakage rate exceeds that of many series [10,13,15], but this discrepancy may be partly explained by our inclusion of closed pseudomeningocele under the definition of CSF fistula. The DP− group had a 3.8% rate of CSF leakage, which we attribute to the “keyhole” incision of the parietal dural leaf. This technical nuance aligns with prior reports emphasizing surgical modifications to minimize leakage [17,18]. Moreover, Tam SKP et al. [15] confirmed in a systematic review and meta-analysis that DP+ carries a higher risk of CSF-related complications; notably, in syringomyelia-positive cohorts, DP+ tended to achieve superior clinical and radiological endpoints, underscoring a trade-off rather than a uniform advantage.

Our analysis of age subgroups revealed no significant correlation between age and syrinx regression, suggesting that in adults, age alone may not determine radiological outcome. This differs from pediatric-focused studies, which have shown better responses in younger patients [26,27]. In addition, El-Hajj et al. [27] highlighted that syrinx characteristics can modulate long-term functional recovery on CCOS, a factor that future adult studies should incorporate.

Durability remains a critical question. Although our one-year follow-up supports short-term sufficiency of DP−, long-term data consistently caution against overgeneralization. Lin et al. reported reoperation rates of 31.9% after DP− [9], whereas Tubbs et al. [23] and Alfieri & Pinna [24] observed far lower reoperation rates (0–3.2%) with DP+. Mozaffari et al. [25] similarly confirmed that DP− carries a higher risk of late failure. Taken together, our results should be interpreted as short-term evidence that DP− may suffice in non-syringomyelia patients, while long-term vigilance remains necessary.

Another limitation of our study is that we did not stratify syringomyelia cases by size or extent. Holocord versus focal syrinx may influence outcomes differently, and future research should incorporate this parameter. Furthermore, we did not evaluate intradural pathology as Yu et al. [22] did, which may partly explain differences between our findings and theirs.

Finally, technique-level choices within DP+ may not dramatically alter global outcomes. Moniruzzaman et al. [30] reported that results did not differ substantially across duraplasty configurations and graft types, supporting individualized technical selection (e.g., graft material, durotomy configuration) according to intraoperative findings and patient-specific anatomy. Taken together, our data show that in carefully selected adults without syringomyelia, DP− achieves short-term clinical gains comparable to DP+ while avoiding a higher CSF-event burden, whereas DP+ confers superior radiological endpoints.

### Limitations

This study has several limitations. This single-center, retrospective study used surgeon-determined assignment to DP+ or DP− (guided by morphometry and intraoperative dural pulsatility) without multivariable or propensity adjustment, so residual confounding is likely. Follow-up was limited to 12 months, precluding inferences about durability, late reoperation, or delayed radiological change. Imaging endpoints warrant caution: the composite “radiological benefit” combines heterogeneous markers; cerebellar slump was defined by a McGregor-based morphometric threshold without external validation; cine phase-contrast CSF was unavailable; and MRI protocols evolved over time. Complication accounting intentionally included closed pseudomeningocele within CSF-related events, which may overestimate leak rates relative to series reporting overt leaks only, while minor events may be under-ascertained. Phenotyping was incomplete (syrinx morphology/size and intradural anomalies not captured), the cohort was underpowered for small effects, and foramen magnum expansion did not track with CCOS. Subgroup analyses (e.g., the syrinx-positive cohort) were exploratory, and the study was not powered to detect small between-group differences in CCOS at one year; longer follow-up may reveal divergence.

Accordingly, generalisability is best limited to adults treated in a tertiary setting using dura-splitting DP− and variable grafts for DP+. Additionally, as a retrospective study, there is an inherent variability in surgical techniques that cannot be fully standardized, despite being performed within a single institution. Subtle differences in decompression extent, dural handling, and surgeon preference may have influenced the observed outcomes. Furthermore, radiological assessments may be subject to interobserver variability, which could affect the interpretation of morphometric measurements and post-operative findings.

## 5. Conclusions

In adults with Chiari I, bone-only decompression achieved short-term clinical improvement comparable to duraplasty, even though duraplasty conferred clearer radiological change and a higher burden of CSF-related events and cerebellar slump. These findings support an individualized strategy that prioritizes symptom profile, syringomyelia status, age, and morphometric context rather than a uniform preference for duraplasty. For carefully selected adults without syringomyelia, bone-only decompression appears clinically sufficient in the first post-operative year, while duraplasty may be reserved for cases in which radiological objectives predominate. Longer, prospective follow-up—ideally stratifying syrinx morphology and intradural pathology—is needed to refine indications and confirm durability. These findings further suggest that surgical decision-making should be guided primarily by clinical presentation rather than radiological parameters alone.

### 5.1. Future Directions and Recommendations

To better isolate the effect of duraplasty, future studies should be prospective, multicenter, pre-registered, and use ≥5-year follow-up with blinded outcome adjudication, preferably randomized or propensity-matched with adjustment for surgeon/center clustering. Imaging must be standardized across sites/timepoints, incorporating cine phase-contrast CSF and quantitative syrinx morphometrics. Stratification should include syringomyelia phenotype (holocord vs. focal; length/diameter; syrinx/cord ratio), tonsillar descent, and posterior fossa morphometry. Outcomes should center on CCOS (primary) with complementary PROMs, reoperation, CSF-related events, and cost-effectiveness. Technique reporting (durotomy configuration, graft material, sealants, dura-splitting variants) should be explicit to enable meaningful technique-level comparisons.

### 5.2. Strengths and Novelty of This Study

This adult, consecutive cohort with complete one-year clinical/MRI follow-up uses a disease-specific outcome (CCOS) and a standardized operative protocol, introduces explicit morphometric definitions (including cerebellar slump relative to McGregor’s line), and demonstrates an anatomy–function dissociation whereby foramen magnum expansion does not track with clinical recovery—together providing a transparent, reproducible framework that clarifies technique trade-offs and refines patient selection beyond radiological thresholds.

## Figures and Tables

**Table 1:** Demographic and clinical characteristics of patients.

Features	
**Gender** **—** **n(%)**	
Female	59 (63.4)
Male	34 (36.6)
**Age** **—** **Min.** **–** **Max (Average ± ss)**	13–70 (34.53 ± 13.51)
**Syringomyelia** **—** **n(%)**	
No	43 (46.2)
Yes	50 (53.8)
**Herniation Degree** **—** **n(%)**	
Grade 1 (5–9 mm)	36 (38.7)
Grade 2 (10–14 mm)	39 (41.9)
Grade 3 (15–19 mm)	14 (15.1)
Grade 4 (>19 mm)	4 (4.3)
**Applied Surgical Method**	
DP+	39 (41.9)
DP−	54 (58.1)
**Pre-operative Clinic**	
Head and neck pain	63 (67.7)
Numbness in extremities	38 (40.9)
Dizziness	15 (16.1)
Difficulty swallowing	12 (12.9)
Loss of balance	10 (10.8)
Loss of strength in extremities	6 (6.5)
Ringing in the ears	3 (3.2)

(Abbreviations: DP+, duraplasty; DP−, decompression without duraplasty ).

**Table 2:** Comparison of post-operative magnetic resonance imaging (MRI) findings at the end of the first year according to the preferred surgical method (DP− vs. DP+).

	DP+		DP−		χ^2^	*p*
	No	Yes	No	Yes		
Syringomyelia	14 (35.9)	25 (64.1)	29 (53.7)	25 (46.3)	2.888	0.089
1st year MCM	14 (35.9)	25 (64.1)	41 (75.9)	13 (24.1)	15.015	<0.001
Decrease in Syringomyelia	10 (40.0)	15 (60.0)	12 (48)	13 (52.0)	16.896	0.000
Cerebellar Slump	27 (69.2)	12 (30.8)	49 (90.7)	5 (9.3)	7.014	0.008
Tonsillar Herniation Reduction	16 (41.0)	23 (59.0)	28 (51.9)	26 (48.1)	1.065	0.302

(Abbreviations: DP+, duraplasty; DP−, decompression without duraplasty; MCM, mega cisterna magna).

**Table 3:** Association Between MCM Formation and Clinical Outcome.

			Clinical Benefit		Total	*p*
			No	Yes		
**MCM in 1st year Magnetic Resonance Imaging**	**No**	**n**	5	50	54	0.71
		**%**	9.1%	90.9%	100.0%	
	**Yes**	**n**	2	36	38	
		**%**	5.3%	94.7%	100.0%	
**Total**		**n**	7	86	93	
		**%**	7.5%	92.5%	100.0%	

(Note: Values are presented as number (%). Statistical analysis was performed using Fisher’s exact test due to small expected frequencies in contingency table cells. The difference between groups was not statistically significant (*p* = 0.71). Abbreviations: MCM: mega cisterna magna).

**Table 4:** Comparison of MRI benefit, CSF fistula development, complications, and clinical benefit according to the preferred surgical method.

	DP+		DP−		Test	*p*
	No	Yes	No	Yes		
**Radiological Benefit**	14 (35.9)	25 (64.1)	41 (75.9)	13 (24.1)	**χ^2^**	<0.001
**CSF Fistula Development**	32 (82.1)	7 (17.9)	52 (96.3)	2 (3.7)	Fisher	0.032
**Complication**	30 (76.9)	9 (23.1)	45 (83.3)	9 (16.7)	**χ^2^**	0.440
**Clinical gain**	4 (10.3)	35 (89.7)	3 (5.6)	51 (94.4)	Fisher	0.448

(Note: Values are presented as n (%). Pearson’s chi-square test was used when expected cell counts were adequate; Fisher’s exact test was used when expected cell counts were small. DP+, decompression with duraplasty; DP−, decompression without duraplasty; CSF, cerebrospinal fluid. Abbreviations: DP+, duraplasty; DP−, decompression without duraplasty; CSF, cerebrospinal fluid).

**Table 5:** (**A**) Comparison of CCOS by surgical technique (DP+ vs. DP−). (**B**) Comparison of CCOS by syringomyelia status.

(**A**)					
**Outcome.**	**Group**	**N**	**Mean ± SD**	**Median (IQR)**	***p***
CCOS	With duraplasty (DP+)	39	13.00 ± 2.55	13 (11–15)	0.129 *
	Without duraplasty (DP−)	54	13.77 ± 2.31	14 (12–15)	
(**B**)					
**Outcome**	**Group**	**N**	**Mean ± SD**	**Median (IQR)**	***p***
CCOS	No syringomyelia	39	13.79 ± 2.09	14 (12–15)	0.215 *
	Syringomyelia present	54	13.16 ± 2.67	13 (11–15)	

(Notes: Values are presented as mean ± SD and median (IQR). An independent-samples *t*-test was used; a Mann–Whitney U test was additionally performed due to the ordinal nature of CCOS, yielding similar non-significant results. Two-sided *p*-values are reported. * Mann–Whitney U test. Abbreviations: CCOS, Chicago Chiari Outcome Scale; SD, standard deviation).

**Table 6:** Morphometric Analysis of Foramen Magnum Expansion.

Group	Pre-Operative FM Diameter (mm)	Post-Operative FM Diameter (mm)	Change (Δ)	*p*-Value
DP− (n = 54)	17.8 ± 2.4	22.3 ± 2.6	+4.5 ± 1.1	<0.001
DP+ (n = 39)	18.0 ± 2.2	23.1 ± 2.5	+5.1 ± 1.3	<0.001
Total (n = 93)	17.9 ± 2.3	22.6 ± 2.5	+4.7 ± 1.2	<0.001

(Abbreviations: FM, foramen magnum; DP+, duraplasty; DP−, decompression without duraplasty).

**Table 7:** Correlation Between Foramen Magnum Diameter and 1-Year Functional Outcome (CCOS).

Variable	r (Pearson)	95% CI	*p*-Value
Pre-operative FM Diameter vs. CCOS	−0.041	−0.24–0.16	0.696
Post-operative FM Diameter vs. CCOS	0.017	−0.19–0.22	0.872

(Note: Correlations with CCOS were assessed using Spearman’s rank correlation coefficient due to the ordinal nature of CCOS. Abbreviations: FM, foramen magnum; CCOS, Chicago Chiari Outcome Scale).

**Table 8:** Comparative Analysis of Key Studies on CM-I Surgical Outcomes (Chronological Order).

Study	N	DP− (%)	DP+ (%)	Clinical Benefit	Syrinx Regression	Complication Rate	Reoperation Rate	Key Finding
Deng et al. (2015) [12]	152	0%	100%	82.9%	65.2%	-	-	Small bone DP+ good result
Gürbüz et al. (2015) [8]	39	46.1%	53.9%	DP−: 61.1%, DP+: 81%	DP−: 12.5%, DP+: 92.3%	DP−: 5.6%, DP+: 28.6%	DP−: 22.2%, DP: 14.3%	DP+ more effective in SM and TH > 10 mm; higher complications
Lin et al. (2018) [9]	Meta	-	-	DP−: 80%, DP+: 85%	DP−: 75%, DP+: 83%	DP+: 10–15%	DP−: 2–12%, DP+: 2–3.5%	DP+ better syrinx, more risk
Butensky et al. (2020) [6]	178	44%	56%	~80%	DP−: 62%, DP+: 93%	Higher in DP+	Equal	DP better for syrinx, more complications
Chang et al. (2021) [10]	Meta	-	-	DP−: 80%, DP+: 85%	DP−: 70%, DP+: 85%	DP+: 10–15%	DP−: 5–10%, DP+: 2–5%	DP+ better syrinx, DP− fewer risks
Akbari et al. (2022) [13]	1000+	-	-	DP−: 80.8%, DP+: 89.6%	DP−: 26.9%, DP+: 43.7%	DP−: 13.7%, DP+: 24.3%	DP−: 17.9%, DP+: 8.3%	DP+ better clinical and syrinx, DP− safer
Rodríguez-Blanque et al. (2023) [2]	100+	0%	100%	>90%	80–90%	-	-	DP+ high success, esp. early surgery
Moniruzzaman et al. (2024) [30]	93	6.5%	93.5%	84.9%	89.2%	34.4%	14%	No long-term advantage for DP+
Current Study (2025)	93	46.2%	53.8%	DP−: 94.2%, DP+: 89.7%	DP−: 25.0%, DP+: 64.1%	DP−: 2.3%, DP+: 14.0%	None reported	DP− sufficient in non-syrinx patients; lower complications

(Abbreviations: SM, syringomyelia; TH, tonsillar herniation; DP+, duraplasty; DP−, decompression without duraplasty).

## Data Availability

The datasets generated and/or analyzed during the current study are available from the corresponding author upon reasonable request.

## Abbreviations

CCOS—Chicago Chiari Outcome Scale, CM—Chiari Malformation, CM-I—Chiari Malformation Type I, CSF—Cerebrospinal Fluid, DP+—Duraplasty, FM—Foramen Magnum, MRI—Magnetic Resonance Imaging, MCM—Mega Cisterna Magna.

## References

[1] Visocchi M., Signorelli W.F., Alves Ó.L., Goel A., Parthiban J., Baeesa S., Sharif S., Sampaio F., Ali S.B., Lee J.H. (2025). Indications for surgery and surgical options in Chiari malformation: WFNS spine committee recommendations. Spine.

[2] Rodríguez-Blanque R., Almazán-Soto C., Piqueras-Sola B., Sánchez-García J.C., Reinoso-Cobo A., Menor-Rodríguez M.J., Cortés-Martín J. (2023). Chiari syndrome: Advances in epidemiology and pathogenesis: A systematic review. J. Clin. Med..

[3] Heiss J.D., Patronas N., DeVroom H.L., Shawker T., Ennis R., Kammerer W., Eidsath A., Talbot T., Morris J., Eskioglu E. (1999). Elucidating the pathophysiology of syringomyelia. J. Neurosurg..

[4] Mueller D.M., Oro’ J.J. (2004). Prospective analysis of presenting symptoms among 265 patients with radiographie evidence of Chiari malformation type I with or without syringomyelia. J. Am. Acad. Nurse Pract..

[5] Strahle J., Muraszko K.M., Garton H.J., Smith B.W., Starr J., Kapurch J.R., Maher C.O. (2015). Syrinx location and size according to etiology: Identification of Chiari-associated syrinx. J. Neurosurg. Pediatr..

[6] Butensky S., Rodgers S., Baron S., Schneider S., Mittler M. (2020). Comparison of surgical outcomes in patients with Chiari Type I malformation receiving posterior fossa decompression with and without duraplasty. Child's Nerv. Syst..

[7] Chotai S., Medhkour A. (2014). Surgical outcomes after posterior fossa decompression with and without duraplasty in Chiari malformation-I. Clin. Neurol. Neurosurg..

[8] Gurbuz M.S., Karaaslan N., Caliskan T., Unal E., Berkman M.Z. (2015). Comparison of the Surgical Results for Foramen Magnum Decompression with and without Duraplasty in Chiari Malformation Type 1. Turk. Neurosurg..

[9] Lin W., Duan G., Xie J., Shao J., Wang Z., Jiao B. (2018). Comparison of results between posterior fossa decompression with and without duraplasty for the surgical treatment of Chiari malformation type I: A systematic review and meta-analysis. World Neurosurg..

[10] Chang T.-W., Zhang X., Maoliti W., Yuan Q., Yang X.-P., Wang J.-C. (2021). Outcomes of dura splitting decompression versus posterior fossa decompression with duraplasty in the treatment of Chiari I malformation: A systematic review and meta-analysis. World Neurosurg..

[11] Lu V.M., Phan K., Crowley S.P., Daniels D.J. (2017). The addition of duraplasty to posterior fossa decompression in the surgical treatment of pediatric Chiari malformation Type I: A systematic review and meta-analysis of surgical and performance outcomes. J. Neurosurg. Pediatr..

[12] Deng X., Yang C., Gan J., Wu L., Yang T., Yang J., Xu Y. (2015). Long-term outcomes after small-bone-window posterior fossa decompression and duraplasty in adults with Chiari malformation type I. World Neurosurg..

[13] Akbari S.H.A., Yahanda A.T., Ackerman L.L., Adelson P.D., Ahmed R., Albert G.W., Aldana P.R., Alden T.D., Anderson R.C., Bauer D.F. (2022). Complications and outcomes of posterior fossa decompression with duraplasty versus without duraplasty for pediatric patients with Chiari malformation type I and syringomyelia: A study from the Park-Reeves Syringomyelia Research Consortium. J. Neurosurg. Pediatr..

[14] Antkowiak L., Tabakow P. (2021). Comparative assessment of three posterior fossa decompression techniques and evaluation of the evidence supporting the efficacy of syrinx shunting and filum terminale sectioning in Chiari malformation type I. A Syst. review and network meta-analysis. World Neurosurg..

[15] Tam S.K.P., Brodbelt A., Bolognese P.A., Foroughi M. (2021). Posterior fossa decompression with duraplasty in Chiari malformation type 1: A systematic review and meta-analysis. Acta Neurochir..

[16] Ciaramitaro P., Massimi L., Bertuccio A., Solari A., Farinotti M., Peretta P., Saletti V., Chiapparini L., Barbanera A., Garbossa D. (2022). Diagnosis and treatment of Chiari malformation and syringomyelia in adults: International consensus document. Neurol. Sci..

[17] Mohanty A. (2019). Chiari I malformation: Surgical considerations. Neurol. India.

[18] Yilmaz A., Kanat A., Musluman A.M., Çolak İ., Terzi Y., Kayacı S., Aydin Y. (2011). When is duraplasty required in the surgical treatment of Chiari malformation type I based on tonsillar descending grading scale?. World Neurosurg..

[19] Pepper J., Elhabal A., Tsermoulas G., Flint G. (2021). Symptom outcome after craniovertebral decompression for Chiari type 1 malformation without syringomyelia. Acta Neurochir..

[20] Giammattei L., Messerer M., Daniel R.T., Aghakhani N., Parker F. (2017). Long-term outcome of surgical treatment of Chiari malformation without syringomyelia. J. Neurosurg. Sci..

[21] Beretta E., Vetrano I.G., Curone M., Chiapparini L., Furlanetto M., Bussone G., Valentini L.G. (2017). Chiari malformation-related headache: Outcome after surgical treatment. Neurol. Sci..

[22] Yu Q.-S., Li T., Wan M., Zhang L., Qiao G.-Y., Yu X.-G., Yin Y.-H. (2025). Clinical outcome of different surgical approaches for symptomatic Chiari malformation without syringomyelia: A 13-year retrospective study. Neurosurg. Rev..

[23] Tubbs R.S., Beckman J., Naftel R.P., Chern J.J., Wellons J.C., Rozzelle C.J., Blount J.P., Oakes W.J. (2011). Institutional experience with 500 cases of surgically treated pediatric Chiari malformation Type. I. J. Neurosurg. Pediatr..

[24] Alfieri A., Pinna G. (2012). Long-term results after posterior fossa decompression in syringomyelia with adult Chiari Type I malformation. J. Neurosurg. Spine.

[25] Mozaffari K., Davidson L., Chalif E., Phan T.N., Sparks A.D., Myseros J.S., Oluigbo C.O., Keating R.F. (2021). Long-term outcomes of posterior fossa decompression for Chiari malformation type 1: Which patients are most prone to failure?. Child's Nerv. Syst..

[26] Hale A.T., Adelson P.D., Albert G.W., Aldana P.R., Alden T.D., Anderson R.C., Bauer D.F., Bonfield C.M., Brockmeyer D.L., Chern J.J. (2020). Factors associated with syrinx size in pediatric patients treated for Chiari malformation type I and syringomyelia: A study from the Park-Reeves Syringomyelia Research Consortium. J. Neurosurg. Pediatr..

[27] El-Hajj V.G., Öhlén E., Sandvik U., Pettersson-Segerlind J., Atallah E., Jabbour P., Bydon M., Daniels D.J., Elmi-Terander A., Edström E. (2024). Long-term outcomes following posterior fossa decompression in pediatric patients with Chiari malformation type 1, a population-based cohort study. Acta Neurochir..

[28] Bogdanov E.I., Faizutdinova A.T., Heiss J.D. (2026). MRI-morphometric characterization of Chiari malformation types 0 and 1 with syringomyelia: Implications for diagnosis and pathogenesis. Neurol. Sci..

[29] Massimi L., Frassanito P., Chieffo D., Tamburrini G., Caldarelli M. (2019). Bony decompression for Chiari malformation type I: Long-term follow-up. New Trends in Craniovertebral Junction Surgery: Experimental and Clinical Updates for a New State of Art.

[30] Moniruzzaman S., Kaipainen A., Tervonen J., Huttunen J., Jyrkkänen H.-K., Huuskonen T.J., Rantala S. (2024). Long-term outcome of operated Chiari I patients between 2005 and 2020 in Eastern Finland. Acta Neurochir..

